# Transactions of the American Medical Association

**Published:** 1850-03

**Authors:** 


*Transactions of the American Medical Association.*
Vol. II. pp. 956.

If the merits of a volume were to be judged of by its size, we should at once pronounce these " Transactions " a highly valuable production ; but inasmuch as quality, not quantity, of matter must be the test, and as it often happens that the most voluminous works contain less actual information than those of meaner pretensions, it becomes us to look beyond the cover, into the contents, and see how far this massive volume, may sustain the reputation of the numerous and important body from which it emanates.

We hold that every American physician is more or less interested in the decision of this question.

The " Transactions " represent, in one sense, our medical character. The work is a national one ; emanating from a representative body, supposed to combine within itself, the best talent of the profession in this hemisphere ; which although young in years, has proved itself vigorous and energetic in action.

The best medical minds in America are enrolled amongst its members, and have taken an active part in its proceedings ; and if the Association has failed to make a favorable impression, both at home and abroad, as to the present state of medicine amongst us, it has rather injured the cause of science than promoted it.

It is therefore with some anxiety that we have opened the volume before us ; but as we have proceeded in its examination, doubts and fears have given place to a feeling of satisfaction, that the labors of this great national association have been worthily performed, and that this volume of Transactions, will confer lasting credit upon the medical literature of the United States.

The chief portion of the work consists of the reports of the various committees appointed to investigate the different branches of medicine, and although some of these might, perhaps, with propriety, have been curtailed, or have been confined more strictly to matters connected with the progress of medical science in *this* country ; yet they all manifest a degree of research and an amount of learning highly creditable to those concerned in their preparation.

In connection with these reports, it has been suggested, that the time required in their reading, and the space which they occupy in the printed Transactions, are a sufficient reason for discontinuing them, and a proposition has actually been made to abolish the standing committees altogether. As this matter will come up at the next meeting, to be held at Cincinnati, it deserves the serious consideration of the members. For our own part, we are not prepared for such a sweeping change in the original plan of the Association. Were it adopted, it is to be feared that the time now devoted to the reading of valuable and solid papers, would be consumed in fruitless discussions upon medical politics, and that matters of science, and the true and permament interests of the profession would be overlooked. At the same time we believe that the duties of the standing committees on medical topics should be circumscribed within the limit of the constitution, and their attention directed mainly to new ideas and modes of practice, originating in America.

This would give to their labors a freshness and originality which they do not now possess; while it would impart to the volume of Transactions a more distinctive and national character, and curtail its present unwieldly dimensions.

The first report of the series, on medical sciences, from the pen of Dr. L. P. Yandell, of Louisville, Ky., is a full and elaborate retrospect of the improvements and discoveries in Anatomy, Physiology, Hygiène, General Pathology and Therapeutics, Medical Jurisprudence, Materia Medica, &c., made within the year, so far as these have come to the knowledge of the committee through the medium of medical periodicals, books, &c.; this occupies 82 pages, and is not confined to the progress of medicine in this country alone. Indeed the committee say that they have not "restricted themselves in their retrospect to medicine at home," conceiving that this course would render their report unsatisfactory and incomplete. Whether this extension of the field of enquiry beyond the limits marked out by the rule of the Association, will be generally approved, is perhaps questionable; while at the same time it must be acknowledged, that a very large amount of valuable matter would be excluded, by conforming to the requirements of the law. As it relates to foreign improvements, however, this omission would be less regarded, from the fact that a summary of a like character is to be found in the Retrospects of Braithwaite and

Ranking, which are issued in this country at a cheap rate, and extensively circulated; and hence we cannot fully accord with the views of the committee on this point. It would carry us far beyond the limits assigned to this notice, to examine this interesting report in its various details. As an able and lucid summary of new facts, theories and suggestions, on the several branches embraced in the enquiries of the committee, it will be profitably consulted by the profession, both here and abroad; while the amount of research, industry and taste, displayed in its preparation, will fully sustain the reputation of its accomplished author.

The report of the Committee on Practical Medicine, Dr. D. Francis Condie, chairman, is a clear and concise notice of such epidemic diseases as have occurred in the United States within the year, so far as they have come to the knowledge of the committee. The materials at their disposal did not admit of a full and accurate delineation of many of these diseases, but where they possessed the required information this has been done. Typhus and typhoid fevers, erysipelas in its various grades and types, the febrile exanthemata, dysentery, cerebro-spinal meningitis, yellow fever, and cholera, as they prevailed in different sections of the United States within the year, form the chief topics of this valuable paper. Such annual exhibits of the rise, progress and peculiar characteristics of epidemic diseases, are peculiarly interesting, both for present instruction and for future reference; and we cannot conceive of a more appropriate object for a great national medical association, than to bring this knowledge together in a concise form, and spread it before the world from year to year.

Appended to this report, are several interesting papers on the epidemic diseases of particular localities, written by practitioners who were addressed by the committee. Two of these letters are from New Jersey, one from Dr. J. F. Garrison, of Swedesboro', and the other from Dr. J. Fithian of Bridgeton; the other refers to an epidemic dysentery, as it prevailed in Cambridge, Mass., and is from the pen of Dr. Merrill Wyman, of that place.

Next in order is the report on Surgery. Dr. Nathan R. Smith, of Baltimore, chairman.

Dr. Smith has also confined himself mainly to a retrospect of the progress of surgery in America, within the past year, though he states that " the small amount of the required information, furnished from these sources, (viz., replies to a private circular and the journals,) and the brief period which has elapsed since the last report, will, it is to be hoped, justify the chairman of your committee, in presenting such results as may have fallen within the sphere of his own personal observation ; and also authorize retrospection, in relation to such alleged improvements, as may never yet have been presented to the profession, though of date anterior to the past year.

The employment of anæsthetic agents in surgery, and the comparative merits of ether and chloroform, occupy a prominent place in the report. The dangers to be apprehended from the use of both these agents are fairly stated ; and the following just conclusion is arrived at: "It is a rational inference," says Dr. Smith, "that any agent, sufficiently powerful to render the living system insensible to the pain of a severe surgical operation, (lithotomy, for instance,) must exert a tremendous influence upon the vital powers. It is by virtue of this counter-impression that its remarkable effect is accomplished. Is it not rational, also, to suppose that such powerful impressions will sometimes be injuriously exerted, and that that which is so powerful for good, should occasionally be equally so for evil ? This must be true, if these agents be at all analogous to other articles of the Materia Medica."

As chloroform is the most powerful anæsthetic agent, it is fair to presume that it will prove the most dangerous, when incautiously administered ; and that immediately fatal results from its use will be more numerous. The fifteen fatal cases collected by Dr. Warren, are admitted ; but these are considered a small number in comparison to the millions of subjects to whom it has been administered, and should not be considered, according to the committee, as a valid argument against its use under proper circumstances. "The individual," says Dr. S., "who subjects himself to its influence, ought to feel no more apprehension than he who takes his seat in a rail road car, and much less than one who essays a voyage across the Atlantic."

The superiority of chloroform over ether, is sustained by the testimony of a number of eminent American surgeons, of large experience, whose views were solicited, and altogether from a strong array in favor of this powerful agent. Drs. Warren and Knight, with some other very respectable names, are, however, found against the use of chloroform, and in favor of ether; the former gentleman preferring the chloric ether to either article.

In view of all the facts developed within the year, Dr. Smith fully sustains the views of the former committee, for 1848, in regard to the use of anæsthetic agents, and considers that great progress has been made "in establishing professional and public confidence in these extraordinary agents, and on the vast benefit which is likely to result to mankind, from this achievement of science and humanity."

The remainder of this interesting report is occupied with the history of improvement in the treatment of fractures and dislocations, and with the replies to the circular of the committee upon other practical questions.

They conclude with the sentiment, that "in this department of medicine, the spirit of improvement is abroad in our country, and that the inventive genius which we claim as a national characteristic, is being exercised with signal success, in whatever relates to the science and art of surgery."

We have only to regret that the topics discussed in this valuable paper are confined so exclusively to the mechanical and operative parts of surgery; with so little attention to the higher theme of the medical treatment of surgical diseases. The latter is certainly one of great interest, and we would fain hope that the spirit of progress would be found equally marked in this department of the science.

The report on Obstetrics, from the lively pen of Dr. Gilman, of New York, is divided into two parts, viz., Diseases of Females, and Obstetrics proper.

Under the former head is included, congestions of the os and cervix uteri, and the importance of the speculum in diagnosticating these affections; uterine displacements, with an account of recently invented instruments to correct them, occlusion of the vagina, ovariotomy, &c. And under the latter, the practice of separating the placenta from the uterine wall in cases of placenta previa, and the use of anæsthetics to control the sufferings and facilitate the progress of labor.

Our space will not permit even a passing notice of these several topics, except the last, the importance of which, at this juncture, seems to demand some remark.

There is certainly no question in the whole range of obstetrical practice of equal interest with this.

Under what circumstances should anæsthetic agents be administered to women in the throes of labor? Should they be given in natural labors or only in protracted or instrumental cases? What are the dangers attending them? Have there been fatal results, and if so, how many, and under what circumstances? Which agent is preferable, ether or chloroform, or a combination of the two? These questions are continually presenting themselves, and the great mass of the profession are still in doubt and anxiety in regard to their solution.

While some accoucheurs of high authority have boldly denounced the use of anæsthetics in midwifery altogether, solely on theoretical grounds, and without any personal experience; others of equal credit have, from the first, taken strong ground in their favor, and have given to the profession the results of their extensive experience.

The committee, in entering upon the subject, appear anxious to treat it impartially, and have, we think, presented the profession with a fair and valuable transcript of the present state of the question.

To Dr. Walter Channing, of Boston, they award the credit of having given to the profession the largest number of cases of etherization in midwifery, which have yet been published together, derived not only from his own large experience, but from forty or fifty physicians residing in and around Boston, amounting in all to five hundred and fifteen cases of natural labor, and fifty-two cases of instrumental or complicated labor. The results of this large number of cases are carefully analysed, and are declared to be highly favorable to the use of anæsthetics in natural labor, while in the instrumental and complicated cases, the success has been beyond what is usual, and "most unexpectedly favorable." Dr. Channing's experience on this point, is compared with that of Dr. Collins, of Dublin, whose work "presents the largest mass of statistical information on obstetrics ever collected in the English language," and a difference in favor of Dr. Channing is clearly shown.

Reports from Dr. G. N. Burwell, of Buffalo, Dr. H. Lindsly, of Washington, D. C., and Dr. Joseph Parrish, of Burlington, N. J., are also referred to by the committee, as confirmatory of the favorable estimate of etherization, derived from the experience of Dr. Channing and his Boston contemporaries, while the foreign reports on this subject, from some of the leading accoucheurs of Great Britain, France and Germany, induce the committee to believe "that anæsthetics are gradually gaining favor with the profession everywhere."

The various advantages of etherization beyond the relief of pain, are also freely discussed; amongst which are mentioned the more perfect contraction of the uterus after delivery, and the consequent diminished liability to hæmorrhage. Upon this point there has been, during the year, considerable testimony, but not in the opinion of the committee of such character as to determine its powers. While the agency in relaxing the soft parts, and its harmlessness upon the fœtus, are considered settled.

Some encouraging experience in favor of the remedial powers of anæsthetics in puerperal convulsions, is also given, and the success reported is believed by the committee to establish their character in this dangerous and frightful disease.

In summing up the results of the enquiry, the committee conclude that the cases in which ether or chloroform have been given to parturient women must now be enumerated by thousands, and "in no one case has a fatal result followed."

"This result, (say they,) if it stood alone, would abundantly establish the safety, as well as the power, of anæsthetics, and would enable us to confer upon woman the greatest benefit she has ever received from medical science." In surgery, however, death has in several cases occurred, as the direct effect of the inhalation, and this has had a most decided influence upon anæsthesia in obstetrics. The extent to which these unfortunate results in surgery should operate, is a question which obstetricians must decide for themselves, and upon which the committee decline the expression of a positive opinion. In this connection an ingenious explanation of the different results in surgery and obstetrics, furnished by Dr. Channing, is given.

In surgery, says Dr. C., the agent is used as a preparative to the operation. Pain is not at the time present, and has not yet exerted its influence on the nervous system—that system is in its integrity, and has of course its greatest capacity for impression, the greatest amount of sensibility, with the least power of resistance. The mind, too, consents to the same thing, and no moral resistance is made; whereas, in labor the pain is present, and has been for a long time; the nervous system has been greatly taxed, its power has, so to speak, been used up. Impressions upon it are weaker than they would be under other circumstances—the system comes readily under the anæsthetic influence—very little ether or chloroform is necessary, and when it is used it is never pushed by the judicious accoucheur to the entire destruction of consciousness. Whether this explanation be or be not satisfactory, it is certainly true that all the deaths reported from the use of chloroform have occurred in surgical practice, and in most of them it has been given preparatory to trivial operations.

The committee compare the relative advantages of ether and chloroform, and evidently incline to the latter, believing that it possesses every advantage except safety. They acknowledge, however, that the administration of this article requires a great amount of skill and tact, which can ordinarily be gained only by experience; and hence they recommend that all persons not practically familiar with anæthesia, should make their first trials with ether. Where thorough knowledge and abundant skill exists, then few if any fatal cases will occur. In regard to the limits within which anæsthesia should be practiced in midwifery, the committee remark, that those who resort to it indiscriminately, or in ordinary cases of labor, are in a very small minority; while a very large majority of the profession would favor it in operative midwifery, and perhaps a majority would give either ether or chloroform in difficult cases of complex or of regular labor. In operative midwifery the committee speak with great confidence of the advantages of anæsthesia, believing that the patient's chance of recovery is greatly augmented by the suffering and exhaustion which she is saved.

Several other points are briefly noticed in the report, which we shall pass over, and reserve a notice of the other contents of the volume for our next number.

